# Impact of Dietary Protein on the Management of Pediatric Short Bowel Syndrome

**DOI:** 10.3390/nu15132826

**Published:** 2023-06-21

**Authors:** Igor Sukhotnik, Reut Levi, Hadar Moran-Lev

**Affiliations:** 1 Department of Pediatric Surgery, Dana-Dwek Children’s Hospital, Tel Aviv Sourasky Medical Center, Sackler Faculty of Medicine, Tel Aviv University, 6 Weizmann St., Tel Aviv 6423906, Israel; 2 Department of Pediatric Gastroenterology, Dana-Dwek Children’s Hospital, Tel Aviv Sourasky Medical Center, Sackler Faculty of Medicine, Tel Aviv University, 6 Weizmann St., Tel Aviv 6423906, Israel

**Keywords:** short bowel syndrome, intestinal failure, dietary protein, intestinal adaptation, hydrolyzed formula, amino acid formula, glutamine, citrulline

## Abstract

Essential amino acids (AAs) play a key role in stimulating intestinal adaptation after massive small gut resection. The nutritional effect of dietary amino acids during intestinal regrowth has received considerable attention in recent years. This review explores the significance of dietary amino acids in the nutritional management of infants and children with intestinal failure and short bowel syndrome (SBS) as reported in the medical literature over the last three decades. A literature search was conducted using electronic databases. Breast milk emerged as the first-line enteral regimen recommended for infants with SBS. Hydrolyzed formulas (HFs) or amino acid formulas (AAFs) are recommended when breast milk is not available or if the infant cannot tolerate whole protein milk. The superiority of AAFs over HFs has never been demonstrated. Although glutamine (GLN) is the main fuel for enterocytes, GLN supplementation in infants with SBS showed no difference in the child’s dependence upon parenteral nutrition (PN). Circulating citrulline is considered a major determinant of survival and nutritional prognosis of SBS patients. Early enteral nutrition and dietary supplementation of AAs following bowel resection in children are essential for the development of intestinal adaptation, thereby eliminating the need for PN.

## 1. Introduction

Intestinal failure (IF) covers a variety of pathologies that lead to a decrease in the actual functional gut mass below that which can sustain growth of the body and/or fluid/electrolyte hemostasis, resulting in dependence upon supplemental parenteral nutrition (PN) for a minimum of sixty days within a period of seventy-four consecutive days [1]. Short bowel syndrome (SBS) is one of the major causes of IF [1,2]. Although intestinal remnant length is commonly used to define IF, particularly that associated with SBS, the functional capability of the gut is actually of greater importance. Many centers consider SBS a functional rather than an anatomic entity, and they define patients who require parenteral nutrition (PN) support for >1 to 3 months after massive bowel resection as having SBS [2]. The Canadian Association of Pediatric Surgeons (CAPS) defines SBS as the need for PN for longer than six weeks after massive gut resection or a residual intestinal length <25% expected for GA (gestational age) [3]. SBS is associated with diarrhea, leading to fluid loss and dehydration, electrolyte disturbances, malabsorption, steatorrhea, and progressive malnutrition, requiring PN support for as long as it persists. PN is a life-saving procedure for SBS patients, and the prognosis of SBS has greatly improved in the decades because of its availability [4]. However, continual administration of may result in many complications, including PN-associated liver dysfunctions, central line catheter bloodstream infections (CLCBI), vascular thrombosis, metabolic abnormalities, and organ dysfunction.

Although PN is necessary as the first step, intraluminal nutrients are essential in stimulating intestinal adaptation (IA) and should be started as soon as possible. Enteral feeding (EF) is preferred because it is more physiological and because it exposes the gastrointestinal (GI) tract to nutrient and hormonal stimuli, improves feeding tolerance, and reduces PN duration, thereby decreasing the risk of CLCBI and intestinal failure-associated liver disease (IFALD). Intraluminal nutrients and substrates can protect against stress-induced gastropathy and GI hemorrhage. Enteral nutrition (EN) leads to contraction of the gall bladder and stimulates secretion of pancreatic and gut luminal hormones, which improves digestion and quickens gastrointestinal motility. Finally, EF is more economical, easier to administer, and safer than PN, and its initiation is therefore recommended om the early postoperative stage. Successful implementation of EN and PN can greatly enhance intestinal rehabilitation, decrease the rate of hospitalization, and improve the quality of life of infants and children with SBS [5].

This review aims to expand on previous research and identify key themes related to the administration of dietary proteins and amino acids in infants and children with SBS. A literature search from 1966–2022 was conducted using a range of electronic databases, including PubMed, EMBASE, Medline, Scopus, and Web of Science.

### 1.1. Establishing Enteral Feeding

There are three phases to take into account in the management of SBS when considering appropriate nutrition. The first stage of care (acute phase) typically takes place immediately after the initial intestinal loss or at the time of SBS diagnosis, when post-resection ileus and impaired fluid/electrolyte hemostasis are prevalent. This phase is marked by gastric and/or stool loss, as well as poor absorption of fluid, electrolytes, and almost all nutrients and micronutrients [6]. Aggressive fluid and electrolyte replacement therapy, acid suppression, and PN are necessary to reduce life-threatening complications such as dehydration, hypotension, and fluid/electrolyte imbalances. EN should be initiated when possible, either orally or via a nasogastric (NG) tube. Scolapio and Fleming recommended therapeutic guidelines for the management of these patients [7]. Their guidelines include the replacement of fluid/electrolytes lost through NG suctioning and in stools. Additional fluids should be added to the total volume to replace insensible losses, and daily urine output should be at least 1 mL/kg/day.

The second management stage (adaptation phase) starts after fluid-shift stabilization and lasts from several days to 24 months. During this stage, 90% to 95% intestinal adaptation (IA) is achieved. This is a time of gradual advancement of EN and the provision of protein and energy from PN. At this time point, accurate monitoring of the patient’s nutritional status is critical. Serum electrolytes, protein status (total protein, serum albumin, and total iron-binding capacity), and liver function tests should be performed on a weekly basis. Fat-soluble vitamin levels should be assessed approximately every six months. Serum levels of vitamins A, D, K (indicative of prothrombin time), and E can be assayed. Patients with a significant loss of the terminal ileum should have their vitamin B12 level assessed every three months.

The last stage of nutritional management (the maintenance phase) is the longest and can sometimes continue for several years. This phase involves gradual weaning from PN support and an increase in the intestinal absorptive capacity with gradual advancement in EN. The primary goal of the third stage is to achieve bowel autonomy (BA) while maintaining body growth velocity and weight gain. Attempts should be made to compensate for unceasing malabsorption by implementing an increased number of small meals and supplementation with micronutrients and vitamins. At this point, if the patient has achieved maximal BA, homeostasis (metabolic and nutritional) can be achieved mainly by enteral feeds. If the patient’s absorptive capacity is not sufficient to sustain growth and the patient is devoted to supplemental nutritional support for life, either ambulatory home PN and/or specialized oral or enteral feedings are necessary.

**Figure 1:**
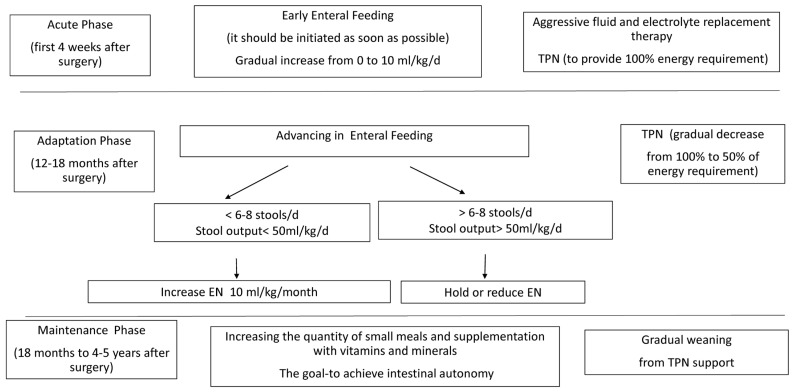
Feeding protocol for infants and children with short bowel syndrome. EN—enteral nutrition, PN—parenteral nutrition.

EN is introduced as soon as signs of food tolerance are observed, starting with small volumes and advancing gradually to the maximal tolerated volume. A combination of bolus and continuous EN is usually provided. Continuous EN may increase mucosal contact time, together with saturation of mucosal transporters and receptors and an overall improved absorption per unit length. However, intermittent feeding, is more physiological, providing a cyclical hormonal surge and promoting regular gallbladder contractions. The introduction of early EN provides a more physiological route of nutrient delivery that stimulates the salivary glands, pancreatic and gastric secretions, gut trophic factors, and the secretion of enteric hormones in response to food boluses. It maintains normal sucking and swallowing functions and decreases the risk of later development of oral aversion [8] (Figure 1).

### 1.2. Types of Enteral Feeding: Amino-Acid-Based Formula Versus Breast Milk

Current enteral nutritional strategies for infants with SBS are mainly experience-based rather than evidence-based (Table 1). 

Breast milk is considered the first-line enteral regimen for infants with SBS because it contains high levels of leukocytes, immunoglobulins (A), nucleotides, GLN, and long-chain polyunsaturated fatty acids (LCPFAs), which, together with other components, can positively influence maturation of the gut immune system [5]. High amounts of immunoglobulins and prebiotic substrates also support the developing neonate’s intestinal microbiota. Despite high lactose levels and relatively low contents of miscellaneous proteins, medium-chain triglycerides (MCT), and iron, breast milk is preferred, as it is well-tolerated in infants with SBS. There are many nutrition-complete formulas available for children with SBS and IF; however, there is no consensus on which is best. Although complex proteins may be superior in stimulating adaptation [9], infants with SBS may have increased bowel permeability and harbor a trend toward the development of food allergies. An association between non-infectious eosinophilic colitis and pediatric SBS has been reported and may support that hypothesis [10].

Therefore, when breast milk is not available, current evidence supports the use of amino acid formulas (AAFs) or extensively hydrolyzed formulas (EHFs; protein size of less than 1500 Daltons) as enteral feeding sources. Table 1 depicts the nutritional composition of human milk, AAFs, and EHFs. Many studies comparing HFs and AAFs were small and retrospective, and there is still no definitive evidence as to which type of formula is better [11,12,13]. The findings of some small case series and case reports show that AAFs are more efficient in decreasing PN requirements [12,13,14]. Bines et al. demonstrated that an AAF improved EN tolerance and reduced the need for PN in four children with SBS who had previously required longstanding PN [12]. De Greef et al. described the beneficial effects of Neocate in four infants with SBS. Those authors recommended an AAF (if breast milk is not an option) as the initial EN for SBS infants, suggesting that it may lead to reductions in PN duration, cost, and complication rates, as well as benefits in terms of patient quality of life [15]. Andorsky et al. reviewed the medical records of 30 neonates with SBS and demonstrated that both breast milk and AAFs play a role in IA and in reduced duration of PN and that they are associated with lowered risk of cholestatic liver disease [16]. However, the only randomized study that compared hydrolyzed and non-hydrolyzed formulas did not report any difference in weight gain, tolerance, or energy expenditure [13] (Table 2).

AAFs are considered the primary option in high-risk neonatal populations (e.g., preterm infants after necrotizing enterocolitis) when the patient has undergone large bowel resection (<25% of predicted bowel remaining) or if the patient has a history of protein intolerance [14]. In a study on preterm infants, Premkumar et al. recommended that the AA supply start on the first postnatal day with at least 1.5 g/kg/d to accomplish an anabolic state, continuing with 2.5–3.5 g/kg/d from the second postnatal day [4]. Protein supply should be accompanied by non-protein intakes of >65 kcal/kg/d and adequate micronutrient intakes. A minimum AA intake of 1.5 g/kg/d is recommended for stable-term infants in order to prevent a negative nitrogen balance, while the maximal AA intake should not exceed 3.0 g/kg/d [17].

No difference has been reported between breast milk and AA-based formula in reducing the duration of PN. Capriati et al. recently published a review of EN in neonates with SBS. This review included 10 clinical studies (822 patients) divided into three cohorts: HF-fed patients, HF + human milk-fed patients, and AA + human milk-fed patients. There was no difference in the rate of enteral adaptation or PN duration among the groups. The authors concluded that human milk should be used in SBS patients when possible and that HFs are just as effective as AA-based formulas in terms of adaptation [18].

As a child with SBS grows, it is possible to incorporate partially hydrolyzed formula and even enriched polymeric formula according to the child’s tolerance. Combinations of whole food (blenderized) formulas have been found to be effective in improving symptoms; however, larger studies are needed in order to fully assess their role in nutritional management of SBS patients [19,20].

### 1.3. Dietary Amino Acids and Intestinal Adaptation

Intestinal adaptation (IA) is the natural process of progressive recovery from IF and SBS by which the intestine increases both its absorptive surface area and functional capacity in an attempt to increase fluid, nutrient, and micronutrient absorption [21]. Although intestinal transplantation has emerged during the last few decades as an appropriate alternative for the treatment of children with SBS, IA remains the only chance for their survival. IA begins within 24–48 h of bowel resection and includes structural (morphologic adaptation) and functional changes (functional adaptation). Structural adaptation is characterized by increased the length and diameter of the bowel, heightened villi, deepening of crypts, and increased enterocyte cell proliferation rates. These changes ultimately result in an increased number of enterocytes and increased absorptive surface area. Functional adaptation results in increased nutrient absorption by isolated enterocytes through upregulation of carrier-mediated transport and modifications of the brush border membrane permeability. Over many decades, a considerable amount of research has been dedicated to the identification of factors that may enhance or accelerate intestinal adaptation. These factors include luminal nutrients and other constituents, hormones, GI secretions, and peptide growth factors [22,23]. There is growing evidence to suggest that exposure to intraluminal nutrients may play a key role in stimulating intestinal adaptation [24]. Therefore, attempting EN as early as possible is recommend by many experts for the management of SBS.

The mechanism by which intraluminal nutrients stimulate IA is unknown. EN works through a number of mechanisms, including stimulation of the mucosal hyperplasia by direct contact between nutrients and epithelial mucosal cells, stimulation of trophic pancreatico-biliary secretions, and stimulation of cell proliferation by trophic GI hormone production. Although EN is one of the main factors in enhancing IA, not all nutrients have similar stimulating effects, with growing evidence suggesting that dietary AAs and proteins, short-chain FAs, and LCFAs are the most effective [25].

#### 1.3.1. Glutamine

Glutamine (GLN) is the most abundant non-essential amino acid. It is produced mainly by muscles and is an important component in many physiologic and biologic processes under normal, healthy conditions. It is vital in the process of mammalian cell proliferation, contributing to protein synthesis and serving as the primary nitrogen donor for purine and pyrimidine synthesis. It is also an influential nutrient for rapidly dividing cells, such as those from the immune system and the gut. GLN, rather than glucose, is the major essential respiratory substrate for gut mucosal cells, accounting for over one-third of the total CO_2_ produced in the gut [26]. It is prominent in the regulation of enterocyte cell cycle regulation and proliferation, the modulation of different inflammatory pathways (such as NF-κB and BMP/ STAT signaling pathways), the maintenance of tight-junction proteins, and the protection of intestinal epithelial cells against apoptosis and cellular stresses [27]. In addition, GLN is important for the development of intestinal subepithelial lymphoid tissue and enhances bowel barrier function. Several experiments in animal models have demonstrated positive effects of GLN supplementation in terms of protecting intestinal mucosa from various types of tissue damage. Dietary GLN supplementation was reported to reduce the generation of proinflammatory cytokines, bacterial translocation, and inflamed lesions in rats with experimental colitis [28]. Oral GLN supplementation ameliorated intestinal mucosal damage and reduced bacterial translocation during radiation-induced gut injury in rats [29]. Injection of GLN alleviated sepsis-induced intestinal inflammatory reactions by modulating gut intraepithelial lymphocytes in mice [30].

The clinical efficacy of GLN supplementation in patients with SBS remains a controversial issue. Although some studies have reported favorable effects, several studies have shown no significant improvement in the parameters of intestinal adaptation, small bowel structural changes, stool loss, or absorption of macro- and micronutrients among those patients [31]. However, the combination of a GLN-enriched diet with various hormones and growth factors was shown to improve intestinal absorptive capacity and reduce the duration of PN in patients with SBS. Several clinical trials have shown that intestinal rehabilitation management, including recombinant human growth hormone (HGH), balanced nutrition support, GLN, and dietary fiber, promotes IA in patients with SBS [32,33]. Wales et al. performed electronic searches to detect publications reporting on the use of HGH with or without GLN for the treatment of patients with SBS. Five clinical trials were included in the review, and the results suggest that patients who were treated with HGH with GLN achieved significant reductions in PN at the three-month follow-up [32]. In a prospective randomized clinical trial including 41 adult patients after massive small bowel resection, Byrne et al. demonstrated that HGH, GLN, and an optimal diet improved nutrient absorption and permitted significantly more weaning from PN [33]. Although this trial was performed in adults with SBS, its results correlate with data from clinical studies in pediatric SBS populations [30]. A combination of a glucagon-like peptide 2 analog (teduglutide) and a glutamine-supplemented diet to promote intestinal growth in adults with SBS was discussed in [34].

#### 1.3.2. Arginine

Arginine (ARG) and its precursor, citrulline (CIT), are necessary substrates in humans as intermediates of the urea cycle, as well as substrates for nitric oxide (NO) production. Their supplementation has been investigated for the treatment of various endothelial-dysfunction-related pathologies, including pulmonary hypertension, arteriosclerosis, arterial hypertension, erectile dysfunction, some mitochondrial disorders, and necrotizing enterocolitis [35]. ARG has a relatively high rate of extraction in the intestine and the liver (where ARG is metabolized to ornithine and urea by arginases 1 and 2, respectively). Marealle et al. demonstrated that 0.2 g/kg/day supplementation of ARG and CIT increased total body NO creation in children suffering from sickle cell disease [36]. Furthermore, oral ARG supplementation may result in dose-dependent GI distress, causing increased activity and bioavailability of CIT compared to ARG. Lancing et al. recently demonstrated that intestinal resection in neonatal piglets affected whole-body ARG synthesis but that the resected animals exhibited increased ARG synthesis after adjusting for absolute small intestinal length [37]. In another experiment, Jiang et al. showed that continuous oral supplementation of L-ARG can stimulate IA after gut resection in rats [38]. Further clinical trials are required to determine whether ARG may stimulate IA, improve nutrient absorption, and reduce PN duration.

#### 1.3.3. Citrulline

L-citrulline (CIT) is a non-essential AA that is synthesized de novo in the intestinal epithelium (predominantly in the duodenum and jejunum) from precursor AAs (mainly glutamine and ornithine) derived from either dietary proteins or systemic AA circulation. It is then released across the basolateral membrane of enterocytes into portal circulation [39]. Because CIT is poorly consumed by hepatocytes, it bypasses the liver metabolism and enters systemic circulation. Circulating CIT is therefore considered a biomarker of enterocyte mass and intestinal integrity [40]. Reduced plasma CIT levels represent impaired intestinal function, and they have been identified in various gastrointestinal disorders, including necrotizing enterocolitis, SBS, celiac disease, and chemotherapy- and/or radiotherapy-induced intestinal damage [41]. CIT is considered a major determinant of patient survival and nutritional prognosis in infants and children with SBS [42]. Patients with a plasma CIT level of 20 µmol/L or less have short bowel segments and usually require permanent home TPN. A cutoff level of 15 mmol/L was found to be an accurate prognostic parameter for the likelihood of future PN independence. In addition, CIT has been described as a measure of improved intestinal absorption capacity after intestinal lengthening procedures, as well as after treatment with teduglutide (glucagon-like peptide 2) [43].

Several pharmacokinetic studies have demonstrated that oral CIT supplementation results in a dose-dependent increases in plasma CIT, ARG, and ornithine levels [44]. CIT supplementation was shown to be safe in children. Cox et al used 0.1 mg/kg/day in supplementary food [45], while Silvera Ruiz administered 3 g/m^2^/day for long-term supplementation (four months) [46]. Filippi et al. recently reported that dietary supplementation with CIT improved nutritional status, preserved muscle trophicity, and enhanced intestinal adaptation in a dose-dependent manner after massive intestinal resection in rats [47]. In addition to its prognostic value, recent evidence suggests that CIT supplementation may be useful in enhancing IA after bowel resection. Jirka et al. demonstrated that oral CIT supplementation enhanced CIT and ARG bioavailability after bowel resection, although the intestinal adaptation effects of oral CIT on total body protein metabolism were minor in the late phase [48].

#### 1.3.4. Protein in Solids

The WHO recommends the addition of complementary food at six months of age alongside breast feeding [49]. There are no current data supporting the need to change the timing of exposure to solid foods in infants with SBS. Moreover, intraluminal nutrients are known to have a stimulating effect on proliferation of the gut epithelial cells, the production of trophic hormones, the stimulation of biliary, and pancreatic secretion. In addition, enteral feeding is important in preventing mucosal hypotrophy, inhibition of the intestinal submucosal immune system, and loss of gut barrier function [50]. Therefore, it follows that the digestion of nutrients is important to gut adaptation. The more complex the digestion of a nutrient, the greater the hyperplasia it causes [51]. There are no specific recommendations concerning the amount or type of protein in solid foods for infants with SBS. In general, total proteins are preferred to provide increased assignment to intestinal digestion and absorption. GLN is the main fuel for enterocytes, and it is thought to enhance mucosal hyperplasia. However, supplementation with GLN in infants with different GI diseases did not accelerate PN independence, nor did it improve food tolerance [52]. Although functional drinks and supplements with ARG and CIT are claimed to enhance athletic performance and are therefore popular among both elite and recreational athletes, their role in achieving gut autonomy in children with SBS is unclear.

## 2. Conclusions

The nutritional management of infants and children with SBS is challenging. The effect of EN on IA is important to consider in the development of feeding strategies for such patients. Breast milk is currently the first-line enteral regimen for infants with SBS despite its high lactose content and relatively contents of medium-chain triglycerides and complex proteins. HFs or AAFs have been successfully used when breast milk is not available or if the infant cannot tolerate whole protein milk, although there is no definitive evidence to show which is the better type of formula. The combination of glutamine with growth hormone or glucagon-like peptide 2 analog (teduglutide) and an optimal diet improves nutrient absorption and permits significantly more weaning from PN. However, that treatment modality is still controversial. Although circulating citrulline is considered an accurate prognostic measure of the likelihood of future PN independence, no studies have examined the reciprocal effects of citrulline on gut functions during IA to date. Because EN is a critical element in the management of children with IF and SBS, more large-scale, collaborative studies are needed to investigate the role of dietary proteins to further improve SBS patient outcomes and fill knowledge gaps.

## Figures and Tables

**Table 1:** Macronutrients composition of human milk, amino acid based and extensively- hydrolyzed.

	Breast Milk	AAFs	EHFs
**Per**	100 mL	100 mL	100 mL
**Energy Kcal**	65–70	67	68
**Protein (g)**	0.8–1	1.8	1.88
Protein source	Whey, casein	100% amino acid	Casein
**Fat (g)**	3.5–4	3.4	3.4
Fat source	Triglyceride, linoleic, alpha-linolenic acid	Coconut oil, high oleic sunflower oil, Sunflower oil, canola oil	Coconut oil, linoleic, alpha-linolenic acid
Linoleic acid mg	Fat content is related to maternal diet and weight gain during pregnancy	n/a	530
DHA (mg)	Fat content is related to maternal diet and weight gain during pregnancy	11.3	15.5
**Carbohydrates g**	8	7.2	7.4
Carbohydrates source	Lactose, glucose, HMO	Dried glucose syrup	Dried glucose syrup
**Sugars g**		0.65	0.77

Abbreviation: AAF: amino acid based formula, EHF: extensively-hydrolyzed formula, DHA: Docosahexaenoic acid, HMO: Human milk oligosaccharides.

**Table 2:** Evaluation of the efficacy and safety of dietary protein in children with short bowel syndrome.

Authors	Study Design	Cohort	Formula	Length	Outcomes
Arai et al., 2022 [10]	Case report	SBS child, 51 cm residual gut	Elemental and low residue diet	19 days	Eosinophilic colitis
Goulet et al., 2013 [11]	Systematic review	Systematic reviews, RCTs, meta-analysis of RCTs, Case control/cohort studies, non randomized interventions	BM vs. HF vs. AAF	4–6 months	BM—first choice HF—second choice AAF—third choice
Bines et al., 1998 [12]	Small clinical trial	4 children with SBS (13, 40, 45 and 45 cm of remnant gut)	AAF (Neocate)	48 months (range 39–51 months)	3 patients achieved EA
Ksiazyk et al., 2002 [13]	Prospective, randomized, double-blind study	Ten children with SBS (9 to 75 cm of remnant gut)	HF (Pepti Junior) vs. non-HF mirror formula	60 days	No difference between HF and non-HF on intestinal permeability, weight gain, energy, and nitrogen balance
Shores et al., 2015 [14]	Retrospective study (two groups with or without implementation of EN guidelines)	95 infants with SBS 30 vs. 53 cm of remnant gut	BM as first choice Donor milk if <32 weeks gestation AAF if NEC, large resection, or intolerance	5 years	Implementation of EN guidelines resulted in shorter times to reach feeding goals
De Greef et al., 2010 [15]	Small clinical trial	4 children with SBS (9, 20, 40 and 50 cm of remnant gut)	AAF (Neocate)	3–13 months	All patients achieved EA
Andorsky et al., 2001 [16]	Retrospective medical record review	30 patients with SBS	BM vs. AAF	12 years	Both BM and AAF were positive in reducing PN and cholestatic liver disease

RCT—randomized-controlled trial; SBS—short bowel syndrome; BM—breast milk; AAF—amino acid formula; HF—hydrolyzed formula ; EA—enteral autonomy; EN—enteral nutrition; PN—parenteral nutrition.

## Data Availability

The datasets used and/or analyzed during the current study are available from the corresponding author upon reasonable request.
